# Cryptogenic Organizing Pneumonia Complicated With Cutaneous Disseminated Nocardia Infection: A Case Report and Literature Review

**DOI:** 10.3389/fmed.2022.886056

**Published:** 2022-06-30

**Authors:** Zhijing Wei, Pengchen Bao, Tianle Wang, Wei Wang, Wen-Yang Li

**Affiliations:** ^1^Department of Respiratory and Critical Care Medicine, The First Hospital of China Medical University, Shenyang, China; ^2^China Medical University, Shenyang, China

**Keywords:** cryptogenic organizing pneumonia, cutaneous disseminated nocardia infection, brain abscess, cranial abscess puncture and drainage, Nocardia infection

## Abstract

Nocardia disease is a rare opportunistic infection that usually occurs in individuals with solid organ transplantation, malignant tumors, human immunodeficiency virus (HIV) infection, or chronic lung disease history. Here, we reported a rare case of cryptogenic organizing pneumonia (COP) combined with disseminated Nocardia infection. A 75-year-old man was admitted to the respiratory department due to weakness and poor appetite for 3 months. The chest CT scan showed dense patchy shadows in the dorsal lower lobe of both lungs. After the transbronchial lung biopsy, the histopathological findings supported the diagnosis of COP. During the period of glucocorticoid reduction (oral methylprednisolone tablets 24 mg one time a day), the patient presented with masses on the back and bilateral upper limbs and intermittent fever for 3 days. After admission, the patient underwent a series of examinations and an ultrasound puncture of the mass. The puncture fluid was caseous necrosis, which was confirmed to be Nocardia infection after bacterial culture, so the diagnosis was disseminated Nocardia infection. After 13 days of admission, the patient developed a headache, accompanied by decreased visual acuity and blurred vision. An imaging (enhanced brain CT) examination revealed intracranial space-occupying lesions. The neurosurgeon was consulted and performed transcranial abscess puncture and drainage, intravenous antibiotics (meropenem, etc.) for 2 months, and trimethoprim/sulfamethoxazole (TMP-SMX) for 6 months. The patient was followed up for 3 years and has remained relapse-free. The mortality rate of disseminated Nocardia infection is as high as 85%, especially when combined with brain abscesses. Therefore, timely diagnosis and correct treatment are crucial for the prevention of fatal consequences. The report of this case can enable more patients to receive early diagnosis and effective treatment, so as to obtain a satisfied prognosis.

## Introduction

Nocardia disease is a rare opportunistic infection that usually occurs in individuals with solid organ transplantation, malignant tumors, human immunodeficiency virus (HIV) infection, or chronic lung disease history (1). Cryptogenic organizing pneumonia (COP) refers to organize pneumonia without clear etiology (such as, infection) or other clinical concomitant diseases (such as, connective tissue disease). Long-term glucocorticoid treatment may lead to systemic immunosuppression (2). Here, we reported a rare case of concomitant disseminated Nocardia infection and brain abscess during the glucocorticoid therapy for COP. At the same time, we also reviewed the previous cases of disseminated infections and brain abscesses caused by Nocardia infection, to promote the standardized process of diagnosis and treatment of Nocardia disseminated infections and brain abscesses.

## Case Presentation

**FIGURE 1:**
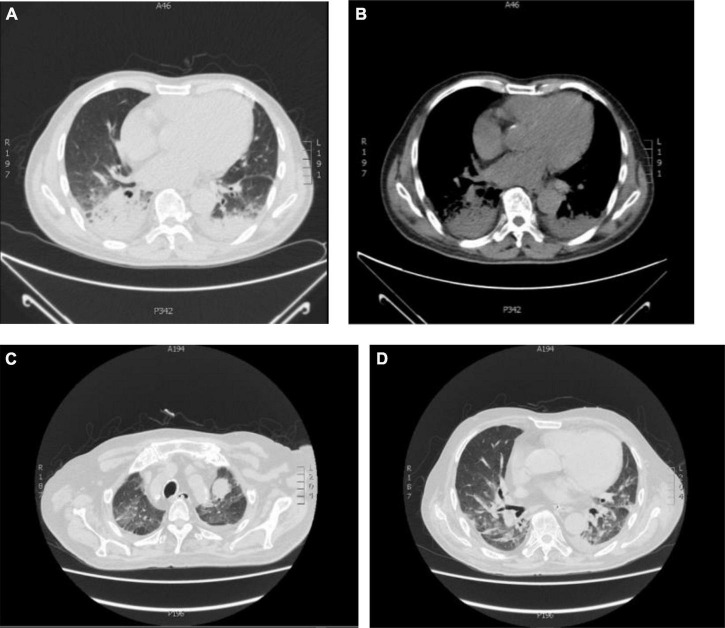
Chest CT imaging findings in two admissions. **(A,B)** Dense patchy shadows in the dorsal segment of bilateral lobes and a small amount of pleural effusion on the right; **(C,D)** chest CT of this admission (the diagnosis of Nocardia infection). Bilateral interstitial inflammatory changes. Old lung and pleura lesions. Limited emphysema in both lungs. Left pleural effusion. Heart enlargement, pericardial effusion, and left neck and left lung apex mass shadow.

**FIGURE 2:**
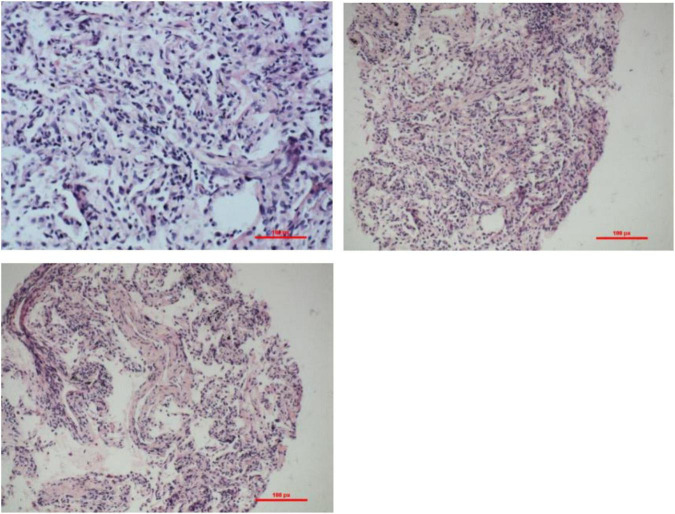
Transbronchial biopsy (TBLB) pathology of the basal segment of the right lower lobe through bronchoscopy: interstitial fibrosis with inflammatory cell infiltration. H&E staining: observed under the light microscope, the alveolar structure was not significantly damaged, alveolar type II epithelial hyperplasia, widening of alveolar space, accompanied by the proliferation of histiocytes and fibroblasts, and infiltrating lymphocytes between the alveolar cavity and small blood vessels.

A year ago, a 75-year-old male patient was admitted to the Department of Respiratory Medicine of the First Affiliated Hospital of China Medical University because of a cough, shortness of breath, fatigue, and loss of appetite. The chest computed tomography (CT) scan showed dense patchy shadows in the dorsal lower lobes of both lungs (Figures 1A,B). The histopathological results supported the diagnosis of COP after completing the transbronchial lung biopsy in our hospital (Figure 2). Then, he was treated with glucocorticoids (40 mg methylprednisolone two times a day intravenously). During the period of glucocorticoid reduction (oral methylprednisolone tablets 24 mg one time a day), the patient was readmitted to the hospital with masses on his back and bilateral upper limbs and intermittent fever for 3 days. At admission, the patientk body temperature was up to 38.5°C without chills and he coughed with a small amount of white sputum. He had poor sleep and diet, stool 1–2 times a day, normal urination, poor mental and physical strength, and a slight weight loss of about 1–2 pounds.

Regarding the past medical history, the patient had cholecystectomy for gallstones for 10 years. He denied any history of hypertension, coronary heart disease, and diabetes.

**FIGURE 3:**
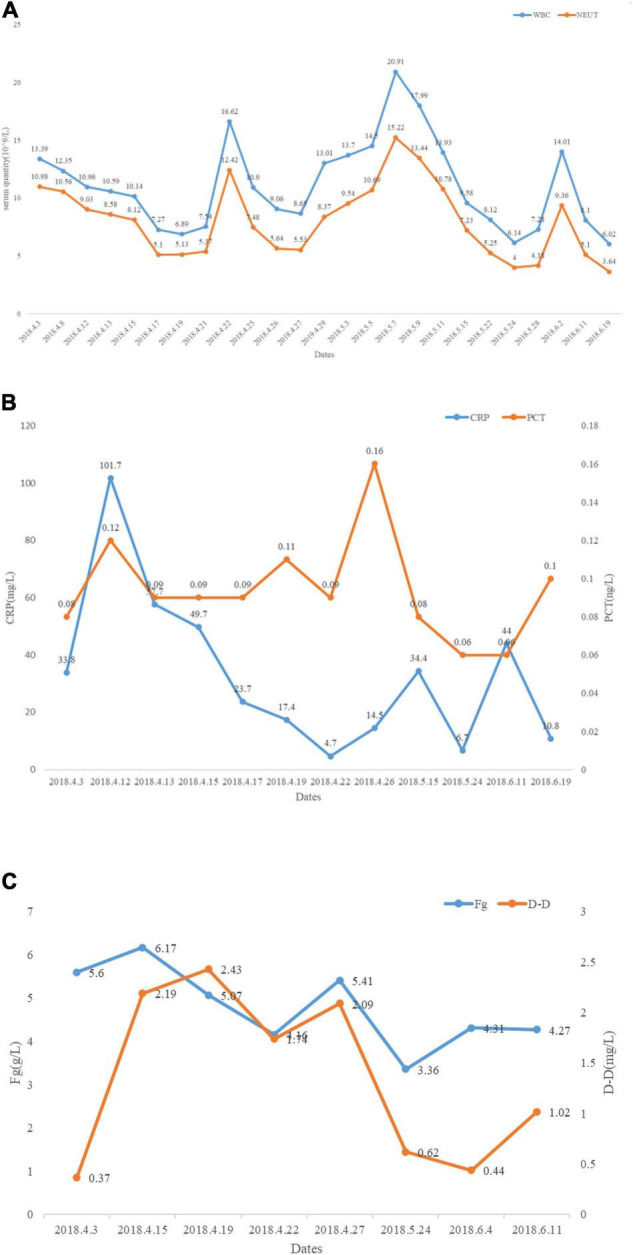
Changes in laboratory study results after taking Piperacillin-Tazobactam, Meropenem, Linezolid, TMP-SMX, etc. **(A)** White blood cell (WBC) and neutrophils (NE) reduced significantly after taking Piperacillin-Tazobactam, Meropenem, Linezolid, TMP-SMX, etc. **(B)** C-reactive protein (CRP) and procalcitonin (PCT) reduced significantly after taking Piperacillin-Tazobactam, Meropenem, Linezolid, TMP-SMX, etc. **(C)** Changes in coagulation indicators during treatment.

**FIGURE 4:**
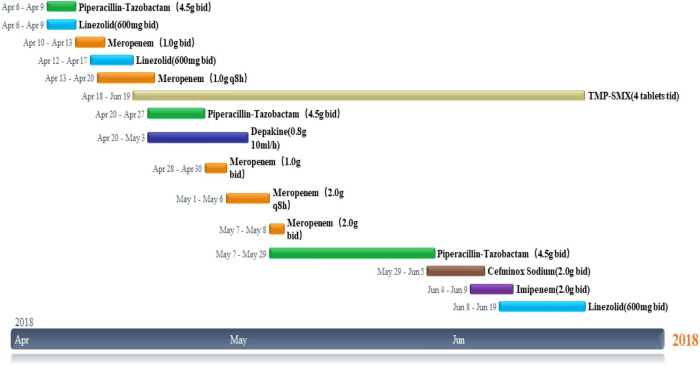
Timeline of drug use during hospitalization.

Laboratory studies showed that the white blood cell (WBC) count was 13.39 × 10^9^/L (normal range 3.5–9.5 × 10^9^/L), absolute neutrophils (NE) count was 10.98 × 10^9^/L (normal range 1.8–6.3 × 10^9^/L) (changes in laboratory study results are shown in Figure 3), C-reactive protein (CRP) 33.80 mg/L (normal range 0–5 mg/L), procalcitonin (PCT) 0.08 ng/ml (normal range 0–0.05 ng/ml) (Figure 3), fibrinogen (Fg) 5.6 g/L (normal range 2–4 g/L), d-dimer assay (D-D) 0.37 μg/ml (normal range 0–0.5 μg/ml) (Figure 3), CD4 T-cell count of 401 cells/μl, and CD8 T-cell count of 212 cells/μl. Blood gas analysis (without oxygen) showed pH: 7.52 (normal range 7.35–7.45), PCO_2_: 27 mmHg (normal range 33–46 mmHg), PO_2_: 88 mmHg (normal range 80–100 mmHg), HCO_3_^–^: 22 mmol/L (normal range 22–27 mmol/L), SaO_2_: 98% (normal range 95–100%). During hospitalization, indexes, such as CEA, CA125, and CA199 revealed no abnormalities. No bacterial fungal growth was observed in both multiple bacterial and fungal cultures. The results of multiple acid resistance staining, T-SPOT and purified protein derivative (PPD), all showed negative. After testing for penicillin allergy, intravenous Piperacillin-Tazobactam (2.5 g every 12 h) and Linezolid (600 mg every 12 h) were initiated for 3 days to anti-inflammation, Eucalyptus, Limoneve, and Pinene (0.3 g every 8 h) and Acetylcysteine tablets (0.6 g every 12 h) for sputum, and active protein and potassium supplementation (Drug use is shown in Figure 4). Considering the obvious side effects of long-term corticosteroid use, corticosteroid therapy was discontinued.

On day 7 post-admission, chest high-resolution computed tomography (HRCT) showed a mass in the left neck and left apex of the lung (Figures 1C,D). Physical examination revealed a palpable mass of about 5 cm in diameter on the left-back, with acceptable mobility, firm texture, and lack of redness, swelling, rupture, heat, and pain. Further improvement of bedside ultrasound showed suspected fibroma lipoma in both upper extremities, and no treatment was needed temporarily; a hypoechoic mass, 5.1 cm × 3 cm, was seen in the left supraclavicular fossa, with irregular shape and strip-shaped blood flow; a fusiform hypoechoic mass was seen in the back area, 2 cm × 1.2 cm, regular shape, uniform echo, can be slightly changed after pressure, strip-shaped blood flow can be seen. The mass on the back is suspected to be caused by infection. It is recommended to take pathology. Meropenem (1.0 g every 12 h) was injected intravenously and Linezolid (600 mg every 12 h) was continued. Meanwhile, for further diagnosis, an ultrasound puncture of the back and neck mass was performed, and 20 ml of caseous necrotic material was taken for antacid bacillus smear and fungal bacterial culture.

On day 10 post-admission, acid-fast bacilli were found to be positive in the puncture smear. Then the time of fungal bacterial culture was further prolonged, samples were incubated at 35°C on Columbia blood agar. At the same time, the patient underwent abdominal enhanced CT scans to exclude other foci, and no obvious abnormalities were found.

**FIGURE 5:**
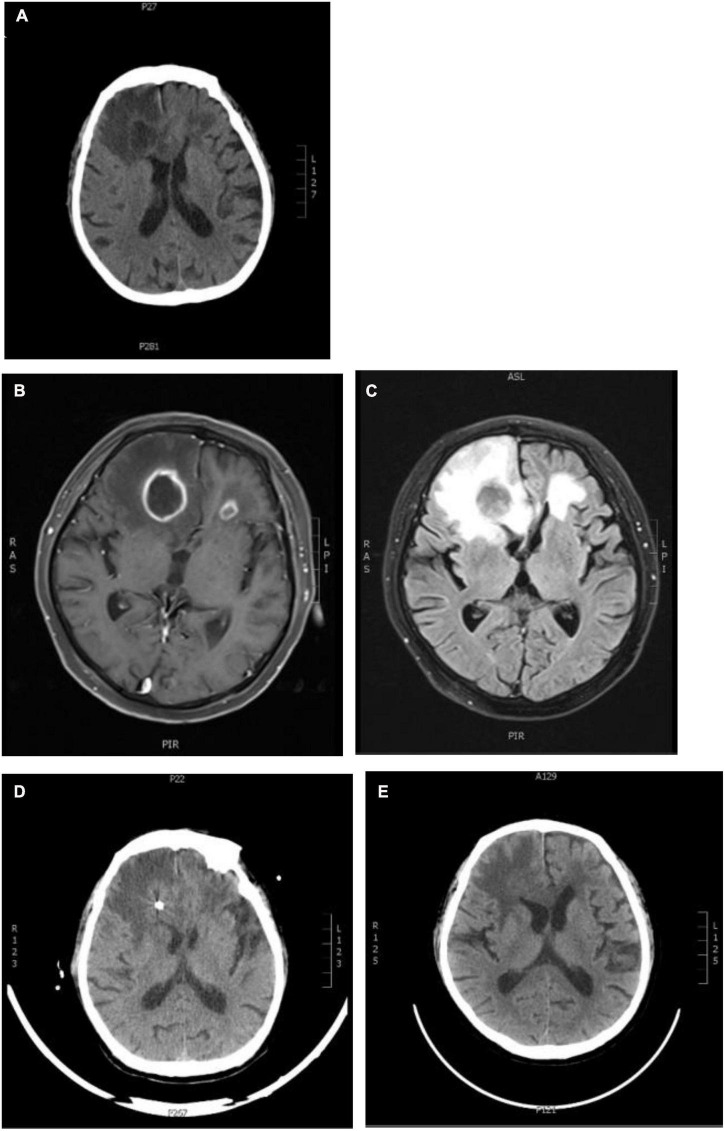
Changes in brain imaging. **(A)** Craniocerebral CT: The patchy low-density shadows were seen in the bilateral frontal and temporal lobes (the right side was the indigenous), and the patchy low-density shadows were also seen in the bilateral basal ganglia and paraventricular zone. The density of white matter in the paraventricular zone was reduced, and there was no abnormal density change in the cerebellum and brainstem; **(B,C)** craniocerebral MRI + enhancement + diffusion: bilateral frontal lobe abscess; **(D,E)** craniocerebral CT after abscess puncture and drainage: postoperative changes, bilateral frontal lobe multiple enhancement lesions, and some lesions shrink.

On day 13 post-admission, the patient suddenly felt a headache, which manifested as throbbing pain in the forehead with nausea, not vomiting, accompanied by vision loss and blurred vision. The cranial CT was completed and showed large infarct foci in the brain (Figure 5A). Further refinement of cranial MR scan + enhancement + diffusion suggested bilateral frontal brain abscess (Figures 5B,C).

**FIGURE 6:**
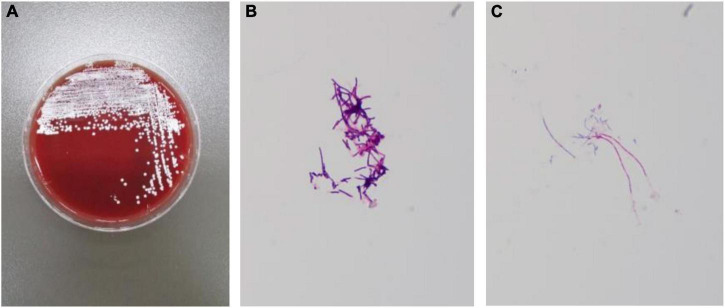
Routine bacterial culture result: Nocardia. **(A)** White, unequal, wrinkling, and granular colonies were growing on the culture medium. **(B)** Gram-staining of bacterial cultures shows Gram-positive bacteria. **(C)** Weak acid-fast staining.

On day 16 post-admission, culture on blood agar grew chalky-white, dry, rough, and raised colonies. The Gram stain of the colonies revealed Gram-positive, branching filamentous bacilli, and staining revealed acid-fast. The bacteria were presumptively identified as belonging to Nocardia species (Figure 6). Antibiotic susceptibility testing was performed by broth microdilution method, in accordance with CLSI M24-A2 guideline (3). Tested antibiotics were gentamicin, penicillin, levofloxacin, teicoplanin, cephazolin, erythromycin, clindamycin, linezolid, fosfomycin, rifampicin, and TMP/SMX. Serial dilutions of each antibiotic were made in a 96-well microplate. Isolates were suspended in 200 μl of sterile water to prepare homogeneous suspensions of the bacteria. After adjustment to 0.5 McFarland standard turbidity, 50 μl of the solution was transferred into 10 ml of Muellerutioton broth and then added to each well of the micro-plate. After 48–72 h of incubation at 37°C, MIC values were calculated and interpreted as susceptible (S), intermediate (I), or resistant (R) according to the CLSI. The results are shown in Table 1.

**TABLE 1:** Summary of antimicrobial susceptibility results of Nocardia.

Antimicrobial agents	Drug sensitivity spectrum characteristics
Gentamicin	S
Penicillin	S
Linezolid	S
Levofloxacin	S
TMP-SMX	S
Teicoplanin	S
Cephazolin	R
Erythromycin	R
Clindamycin	R
Fosfomycin	R
Rifampicin	R

*S, sensitive; R, resistant.*

Following pathological diagnosis and results of antibiotic susceptibility testing, patients were given oral TMP-SMX (4 tablets every 8 h, each piece containing 80 mg of trimethoprim and 400 mg of sulfamethoxazole), and antibiotics were adjusted according to the condition (Figure 3). The patientndisymptoms gradually improved without adverse reactions. Cranial abscess puncture and drainage were performed, intravenous infusion of sodium valproate (0.8 g/10 ml every hour) was given to prevent seizures, and supplemental nutritional therapy was administered. On day 25 post-admission, the patient underwent cranial abscess puncture and drainage in the neurosurgery department, and after the operation, the patient’s symptoms, such as blurred vision and headache improved significantly (Figures 5D,E). The bacterial culture results of the puncture fluid thereafter also confirmed Nocardia infection. After the treatment of Meropenem (1.0 g every 8 h) and TMP-SMX (4 tablets every 8 h) for 11 days (Figure 4), repeat ultrasound showed that all existing skin lesions were absorbed. The blood inflammatory index decreased significantly. With the continuous change of the patiente continuous change of ly.existing skin lesions weradjusted. The patient improved significantly and had no adverse events after 10 weeks. The patient was discharged and recommended to continue taking TMP-SMX for 6 months. Until now (3 years after being discharged), the patient has not relapsed.

## Discussion

Nocardia disease is a rare infection caused by Nocardia. Nocardia widely exists in nature but usually does not exist in human beings. It is common in patients with potential chronic diseases or endogenous or iatrogenic immunosuppression. It can invade the human through the digestive tract, respiratory tract, and skin wounds, causing localized or disseminated suppurative diseases, and the lung is the main affected organ. The mortality rate is 7–44%, and it is higher when Nocardia causes disseminated infection and brain abscess. Therefore, early diagnosis and reasonable treatment are the keys to improve prognosis and reduce dissemination and recurrence (1).

Cryptogenic organizing pneumonia is a kind of organizing pneumonia without definite causes and other accompanying diseases. It is mainly manifested as hyperplasia of granulation tissue in the alveolar duct and alveolar cavity, with mild infiltration of inflammatory cells in pulmonary interstitial and hyperplasia of type II alveolar epithelium. The lung structure of COP is often normal, and the fibrosis caused by COP is reversible. At present, glucocorticoid is the first choice for the treatment of COP, and the general course of treatment is 6d t months. However, COP is more likely to recurrence, glucocorticoid reduction or rapid withdrawal will cause recurrence, and re-use is still effective (2).

The long-term use of glucocorticoid in patients with COP is likely to cause fungal (especially Aspergillus) and bacterial (Staphylococcus) co-infections (4, 5), but there are few cases of COP co-infection with Nocardia (6). We searched relevant literature from 2011 to 2021 in PubMed and Web of Science for COP complicated with other infections, and 9 studies were included (7–15) (Table 2).

**TABLE 2:** Nine cases of fungal, bacterial, and viral infections in patients with COP patients.

Infection type	Study	Application of glucocorticoid (Y/N)	Application time	Outcomes
***Bacteria***				
Actinomycete	Khatib et al. (7)	N	−	Improved
	Alfaro et al. (8)	N	−	Improved
	Fujita et al. (9)	N	−	Improved
Staphylococcus	Gomes et al. (12)	N	−	Improved
Tubercle bacillus	Sander et al. (11)	N	−	Improved
Nocardia	Fernandes et al. (13)	Y	3 months	Improved
***Fungi***				
Aspergillus	Sawai et al. (10)	Y	10 months	Died
	Xie et al. (14)	N	−	Died
***Virus***				
Herpes simplex virus	Cunha et al. (15)	N	−	Improved

This patient was admitted with cough, fever, malaise, and masses in the back and both upper extremities, and imaging showed a mass shadow on the left lung apex and back. According to the patient’s laboratory data, the mass may be caused by infection. However, combined with the previous patient’s medical history, the main differential diagnosis included COP recurrence, common bacterial infections, and special bacterial infections, such as Nocardia. From the analysis of lung imaging, the imaging manifestations of patients with COP are complex and diverse, which can be roughly divided into solid variants, solid nodules/masses, and ground glass (16). Among them, solid deformation is the most common type, the CT manifestation is patchy solid deformation, which is mainly distributed under the pleura or/and along with the bronchial vascular bundle. The bronchial gas phase is seen in some parts, and both sides are common (17). The common imaging manifestations of Nocardia pneumonia are nodule/mass shadow, ground glass shadow, interlobular septal thickening, cavity, consolidation, bronchiolitis, bronchial wall thickening, mediastinal lymph node enlargement, local pleural thickening, and rare air bronchogram. In this case, chest imaging showed bilateral patchy dense shadows at the previous two visits due to COP, and the distribution was mainly subpleural, along with bronchial vascular bundles (18). During this visit, chest CT showed a left unilateral pulmonary apex mass. Although imaging is different from before, it is still necessary to avoid misdiagnosis caused by inertia thinking and delayed treatment.

Since then, a brain abscess was found in the course of treatment, which was manifested as forehead pain, nausea, and other symptoms. Almost at the same time, the culture of skin puncture material suggested that Nocardia was positive, the patient was treated with Meropenem and TMP-SMX promptly, and selective puncture and drainage of brain abscess. In the diagnosis and treatment of Nocardia, the spread of Nocardia affects at least two adjacent organs, especially brain abscess, which is an important factor in the adverse outcome of patients. We searched relevant literature from 2017 to 2021 in PubMed and Web of Science using disseminated Nocardia infection and case reports as the keywords and included confirmed disseminated Nocardia infection involving 2 organs and the above, complete records of adult cases, a total of 126 cases of disseminated Nocardia infection were retrieved, a total of 23 cases were included in the study (13, 19–38), and the remaining 113 cases (58 cases involving less than 2 organs, 23 cases with disease course records incomplete, 12 cases with other infections, 11 cases with no confirmed Nocardia infection, 4 cases where the full text could not be found, and 5 cases reported non-human or adult cases) were excluded. A summary and review of the 23 cases are presented below (Table 3).

**TABLE 3:** Clinical characteristics of patients with Nocardia infection.

Variable	Category	Case, *N* (%)
Sex	Male	20 (87%)
	Female	3 (13%)
Age (mean ± SD)		58 ± 12.15
Immune status	Non-immune compromised	3 (13%)
	Immune compromised	20 (87%)
Comorbidities	Malignancy	2 (8.7%)
	Chronic kidney disease	3 (13%)
	Rheumatic diseases	4 (17.4%)
	Diabetes mellitus	5 (21.7)
	Solid-organ transplant	2 (8.7%)
	Hematologic diseases	2 (8.7%)
	Immunodeficiency disease	1 (4.3%)
	Alveolar protein deposits	1 (4.3%)
	Prednisone (≥ 5 mg/d)	11 (47.8%)
	Immunosuppressive therapy	8 (34.8%)
	Chemotherapy	1 (4.3%)
	Targeting therapy	1 (4.3%)

A total of 126 cases of disseminated Nocardia infection were searched and a total of 23 cases were included in the study. The mean age of onset was 58 years, and 87% of patients were men. The most common comorbidities were diabetes mellitus (21.7%), rheumatism-related diseases (17.4%), and chronic kidney disease (13%). Furthermore, 20 patients were immunocompromised, 11 patients were on glucocorticoid, 9 patients were on immunomodulatory therapy, and 2 patients were on chemotherapy and targeted therapy. Based on statistical analysis, this study hypothesized that corticosteroid treatment is a high-risk factor for disseminated Nocardia infection, which is consistent with previous studies.

Of these cases, 20 patients (86.96%) had pulmonary involvement, 13 (56.52%) had brain involvement, 10 (43.48%) had skin or muscle involvement, and 4 (17.39%) had other areas of involvement, such as the adrenal glands, heart valves, and spine. In total, 23 patients presented with fever, cough, dyspnea, malaise, and weight loss. Among them, 13 patients with brain abscesses presented with fever, cough, dyspnea, headache, and abnormal sensation in the limbs, and one patient presented with new-onset epilepsy. Most patients improved or were cured after prompt treatment, but 6 patients (mortality rate of 26.09%) died due to ineffective treatment or other complications. It is worth noting that among the 6 patients who died, 4 patients were involved the lung and brain at the same time, 3 patients died directly or indirectly from brain abscess and complications (75%), 1 patient discontinued the treatment for unknown reasons, and the specific cause of death was unknown. One of the two other patients without brain involvement died due to ineffective treatment and the other from the primary tumor. Therefore, this study speculates that brain abscesses caused by Nocardia infection may be a high-risk event for poor outcomes and require extensive attention in clinical treatment.

In these cases, TMP-SMX was used in all patients except one patient with meropenem and vancomycin. Currently, sulfonamides are the preferred drugs for Nocardia, but the drug resistance rate has gradually increased in recent years. Patients with sulfonamide allergy, drug resistance, or contraindication can choose amikacin, imipenem, meropenem, ceftriaxone, linezolid, tigecycline, etc. (39). For patients with central nervous system infections, drugs with high blood-brain barrier permeability should be selected, such as ceftriaxone and linezolid. For patients complicated with brain abscesses, in addition to medical treatment, 2 patients underwent surgery, and 4 patients died without surgical intervention, although the death of patients with severe complications, to some extent also triggered our thinking, that surgery will improve the prognosis of brain abscess caused by Nocardia. Previous studies have shown that surgical intervention may be an appropriate method for the treatment of brain abscess, such as abscess puncture drainage and abscess resection. It is generally recommended that abscesses greater than 2.5 cm should be treated with abscess puncture drainage (40).

Previous studies have demonstrated that TMP-SMZ at doses used to prevent Pneumocystis jirovecii pneumonia in patients with hematopoietic stem cell transplant protects against Nocardia infection. However, Steinbrink et al. studied 112 individuals comparing immunocompetent and immunocompromised patients and found that prophylactic use of TMP-SMZ did not provide robust protection against Nocardia infection. Protection against Nocardia infection may be related to the dose of TMP-SMZ and the duration of immunocompromised (41). In cases of COP, glucocorticoids are used for a long time, and cellular immunity is low. In addition to basic measures, such as avoiding a cluttered environment and avoiding contact with high-risk groups of infection to reduce the chance of infection, when we comprehensively consider the patient’s underlying disease and immunity, accepting low-dose TMP-SMX may be an option to prevent Nocardia infection (which remains to be explored), but the diagnosis of Nocardia infection should not be ruled out in individuals receiving TMP-SMX prophylaxis.

In this case, the patient’s long-term oral glucocorticoids increased immunodeficiency, leading to a higher risk of Nocardia infection. We speculated that the most likely route of infection of Nocardia might be inhaling the lungs and causing disseminated infections in the brain and skin through blood transmission. This case has a history of long-term use of glucocorticoids in the treatment of COP, which provides us with thinking. In patients with COP, the new inflammatory changes in the lungs must be vigilant whether COP recurrence or concurrent opportunistic infections, such as Nocardia. This case also provides suggestions for the clinical manifestations and treatment of disseminated Nocardia infection, especially brain abscess, which can enable more patients to receive early diagnosis and effective treatment to obtain a better prognosis.

## Ethics Statement

Written informed consent was obtained from the individual(s) for the publication of any potentially identifiable images or data included in this article.

## Author Contributions

ZW: study design, data analysis, manuscript drafting, and final approval of the version to be published. PB, TW, and WW: providing language help, writing assistance, and final approval of the version to be published. W-YL: study design, data interpretation, critical manuscript revision, and final approval of the version to be published. All authors contributed to the article and approved the submitted version.

## Conflict of Interest

The authors declare that the research was conducted in the absence of any commercial or financial relationships that could be construed as a potential conflict of interest.

## Publisher’s Note

All claims expressed in this article are solely those of the authors and do not necessarily represent those of their affiliated organizations, or those of the publisher, the editors and the reviewers. Any product that may be evaluated in this article, or claim that may be made by its manufacturer, is not guaranteed or endorsed by the publisher.
